# Optimization of methyl orange decolorization by bismuth(0)-doped hydroxyapatite/reduced graphene oxide composite using RSM-CCD

**DOI:** 10.1007/s11356-024-33425-4

**Published:** 2024-04-27

**Authors:** Umit Ecer, Sakir Yilmaz, Berdan Ulas, Serap Koc

**Affiliations:** 1 Department of Chemical Engineering, Institute of Natural and Applied Sciences, Van Yuzuncu Yil University, 65080 Van, Turkey; 2 Department of Mining Engineering, Faculty of Engineering, Van Yuzuncu Yil University, Van, 65000 Turkey; 3 Department of Mechanical Engineering, Faculty of Engineering, Van Yuzuncu Yil University, Van, 65000 Turkey

**Keywords:** Bismuth, Decolorization, Methyl orange, Hydroxyapatite, Reduced graphene oxide

## Abstract

In the current study, the catalyst for the decolorization of methyl orange (MO) was developed HAp-rGO by the aqueous precipitation approach. Then, bismuth(0) nanoparticles (Bi NPs), which expect to show high activity, were reduced on the surface of the support material (HAp-rGO). The obtained catalyst was characterized by scanning electron microscopy (SEM), energy-dispersive X-ray analysis (EDX), X-ray diffraction (XRD), and X-ray photoelectron spectroscopy (XPS) techniques. The parameters that remarkably affect the decolorization process (such as time, initial dye concentration, NaBH_4_ amount, and catalyst amount) have been examined by response surface methodology (RSM), an optimization method that has acquired increasing significance in recent years. In the decolorization of MO, the optimum conditions were identified as 2.91 min, Co: 18.85 mg/L, NaBH_4_ amount: 18.35 mM, and Bi/HAp-rGO dosage: 2.12 mg/mL with MO decolorization efficiency of 99.60%. The decolorization process of MO with Bi/HAp-rGO was examined in detail kinetically and thermodynamically. Additionally, the possible decolorization mechanism was clarified. The present work provides a new insight into the use of the optimization process for both the effective usage of Bi/HAp-rGO and the catalytic reduction of dyes.

**Supplementary Information:**

The online version contains supplementary material available at 10.1007/s11356-024-33425-4.

## Introduction

The primary causes of severe, irreversible environmental damage are indiscriminate urbanization, industrialization, and rapid population growth. Every day, numerous millions of gallons of wastewater are released by industries that produce textiles, refineries, paper pulp, pesticides, batteries, and other products. The terrestrial and aquatic natural environment’s bodies, including rivers, ponds, and lakes, are endangered by the organic pollutants found in effluent. Complicated molecules and organic dyes are used in the printing, tannery, paint, textile, and plastics industries. Presently, over 10 million distinct dyes are produced annually throughout the world (Sen et al. 2011). Because they are not completely fixed, 12–15% of the dyes used in manufacturing processes are released into the effluent (Al-Amrani et al. 2014). Due to the abovementioned problems, scientists have directed their research towards the treatment of organic pollutants in industrial wastewater and the development of innovative treatment methods. Among the treatment techniques for dye-contaminated water, methods such as coagulation (Liu et al. 2022), ion exchange (Joseph et al. 2020), adsorption (Zolfaghari et al. 2023), electrochemical treatment (Bustos-Terrones et al. 2021), flocculation (Januário et al. 2021), and chemical reduction (Truong et al. 2024) have been used recently.

Various studies have been reported examining the degradation of dyes by various reducing agents (Acar et al. 2023; Joseph and Mathew 2015; Osunlaja et al. 2012). The degradation of methyl orange (MO) in the presence of different reducing agents has been studied by many scientists. It is a known fact that reducing agents add electrons or donate hydrogen to the substrate. It should be noted that the reducing agent itself is oxidized during the reduction process (Patel et al. 2022). In the current study, sodium borohydride (NaBH_4_) was chosen as the reducing agent due to its unique ability to reduce imine and carbonyl functional groups to amines and alcohols, respectively. The reaction rate in the decolorization reaction is significantly influenced by the choice of catalyst. Recently, metal nanoparticles (MNPs) have gained attention as catalysts because of their special characteristics. Despite their seeming uniqueness, MNPs tend to collect in clusters because of their high surface energy, which lowers catalytic activity and causes long-term stability issues. A large surface area supporting material can be used to solve the MNP agglomeration problem. To produce MNPs with a controlled size and distribution that are both stable and active, it is important to choose the right support (Ecer et al. 2023). MNPs (Pd, Pt, Ag, Bi, Ni, Co, Cu, etc.) have been extensively investigated because of their mechanical, catalytic, and electrochemical qualities in a variety of fields. Bismuth nanoparticles (Bi NPs) are one type of metal-based catalyst that has intriguing properties for the degradation of pollutants in water. Compounds based on Bi NPs and their composites have a variety of useful uses, such as energy storage, gas sensors, and catalysts. These substances are well known for their low cost, good dielectric qualities, high oxygen conductivity, and non-toxicity (Alovn et al. 2023). However, because of their small size and high surface energy, metal nanoparticles are prone to aggregation, which will decrease their catalytic activity. Moreover, it is challenging to recover and recycle Bi NPs from the aquatic system, which can lead to secondary contamination and restrict their practical uses (Ecer and Yılmaz 2024). Supporting materials have been utilized to immobilize NPs onto solid supports to address such issues. These materials included clay minerals, biochar, silica gel, activated carbon, polymers, and magnetic materials (Khan et al. 2023; Xue et al. 2023).

Graphene oxide (GO), a kind of 2-D nanostructured sp^2^ carbon material, has garnered a lot of attention because of its remarkable qualities, which include high optical transparency, great electrical conductivity, and a vast surface area. Utilizing the graphene nanosheet as an ideal conductive platform for nanoparticles to create hybrid nanocomposites is therefore highly promising. Additionally, it is verified that the graphene nanosheet can stop nanoparticle aggregation (Ding et al. 2021). Compared to GO, reduced graphene oxide (rGO), a chemically altered form of GO, is more economically suitable for large-scale manufacturing. rGO is widely used in the synthesis of GO-based composites. rGO exhibits higher surface area, higher electrical conductivity, superior electrocatalytic properties, and more efficient carrier mobility than GO (Liu et al. 2018; Wei et al. 2017). Additionally, rGO enhances cellular behavior due to its biocompatibility and the presence of a small number of functional groups in its basal plane and edges, including hydroxyl, epoxy, carboxyl, and carbonyl. These groups enable the creation of bioactive nanomaterials with customized microstructures and enhanced mechanical properties. Hydroxyapatite (HAp) is one of the major common forms of ceramic biomaterials. Bio-ceramics are an example component of HAp with carbon (C) nanotubes that have only recently been studied. Because of its excellent biological properties, biocompatibility, biomedical application properties, and bioactive, it is the primary constituent of mammalian hard tissues (tooth and bone); it was also employed for a variety of medical applications. Ca_10_(PO_4_)_6_(OH)_2_ is the main chemical compound with the chemical formula of pure HAp. Pure HAp has a Ca:P atomic ratio of 1.67, indicating large stability and calcium orthophosphates have molecular ratios ranging from 2 to 0.5 (Ciobanu et al. 2015; Ciobanu and Harja 2019). Combining HAp and reduced graphene oxide (rGO) can create a material with several advantages. As a result of doping, the material’s properties such as mechanical, electrical, surface properties and biocompatibility can be improved, making it more effective in various biomedical and material science applications. In particular, the mechanical biocompatibility and performance of HAp can be developed seriously by reinforcement with rGO (Lee et al. 2015).

To maximize the benefits of a system, process, or product, it is necessary to optimize its performance. Finding the optimum conditions to apply a process that yields the best outcome is commonly referred to as optimization. Traditionally, optimization has been done by tracking the impact of a single factor on an experimental response one at a time. Only one parameter is altered; the values of the others remain unchanged. The main drawback of this approach is that it ignores the interactions between the variables under investigation. Consequently, this method does not fully illustrate how the parameter affects the response (Hanrahan and Lu 2006). The increased number of experiments required to carry out the research, which increases time and costs as well as the consumption of reagents and materials, is another drawback of one-factor optimization. Multivariate statistical techniques have been used to optimize analytical procedures to solve this issue (Bezerra et al. 2008). Response surface methodology (RSM) is one of the most pertinent multivariate techniques used in analytical optimization. The goal of response surface methodology is to make statistical predictions by describing the behavior of a data set using a set of mathematical and statistical techniques based on the fit of a polynomial equation to the experimental data. It works well when many factors influence a response or a group of relevant responses. To achieve optimal system performance, the goal is to concurrently optimize the levels of these variables (Bezerra et al. 2008; Hanrahan and Lu 2006).

In the current work, HAp-rGO was first produced using the aqueous precipitation method to develop the catalyst that would be used in the decolorization process. Then, using the NaBH_4_ reduction method, bismuth(0) nanoparticles (NP), which we anticipate to exhibit high activity, were deposited on the surface of the support material (HAp-rGO). RSM was used to model the optimization of the key parameters influencing the decolorization of MO (time, MO concentration, NaBH_4_ amount, and catalyst amount). Maximum decolorization efficiency (%) was then calculated by identifying the parameters optimum points using the resulting model equation. Scanning electron microscopy (SEM), energy-dispersive X-ray analysis (EDX), X-ray diffraction (XRD), and X-ray photoelectron spectroscopy (XPS) analyses were conducted to elucidate the surface properties of the obtained catalyst. Additionally, the decolorization of MO using the Bi/HAp-rGO was examined in terms of thermodynamics and kinetics. Furthermore, the possible mechanism of decolorization was clarified. The current study offers a fresh viewpoint on the use of the optimization procedure for the catalytic reduction of dyes as well as the efficient utilization of Bi/HAp-rGO.

## Material and methods

Pure HAp and HAp-rGO (rGO, purity %99, S.A 15.62 m^2^/g, 2–5 layers, nanografi) composite were synthesized by an aqueous precipitation method [1]. Calcium nitrate tetrahydrate (Ca(NO_3_)_2_4H_2_O), di-ammonium hydrogen phosphate ((NH_4_)_2_HPO_4_), rGO (wt% 5) were used as starting reagents. To prepare the pure and doped samples, Ca(NO_3_)_2_4H_2_O (solution 1) and (NH_4_)_2_HPO_4_ (solution 2) were added into distilled water to prepare the solutions with a certain molar ratio. These two powders were dissolved separately in distilled water with a Ca/P ratio of 1.67. Ammonia (NH_4_OH) was added into (NH_4_)_2_HPO_4_ solution after previous solutions were stirred for 1 h. NH_4_OH was added to both solutions to bring the pH level to 11–12. Different from pure HAp, rGO was added at the same time into the solution in a dropwise manner after stirring for 10 min. The final mixture was heated until boiling to increase the reaction. After boiling, the mixture was left for stirring for 24 h. After 1 day of aging, the solution was filtered to obtain a wet cake. The wet cake was dried in an oven at 200 °C to remove the excess water and ammonia. The precipitated and dried HAps were crushed with an agate mortar and pestle, and the resulting powder and bulks were sintered at 1100 °C for 1 h (Gungor Koc 2019; Song et al. 2001).

**Fig. 1:**
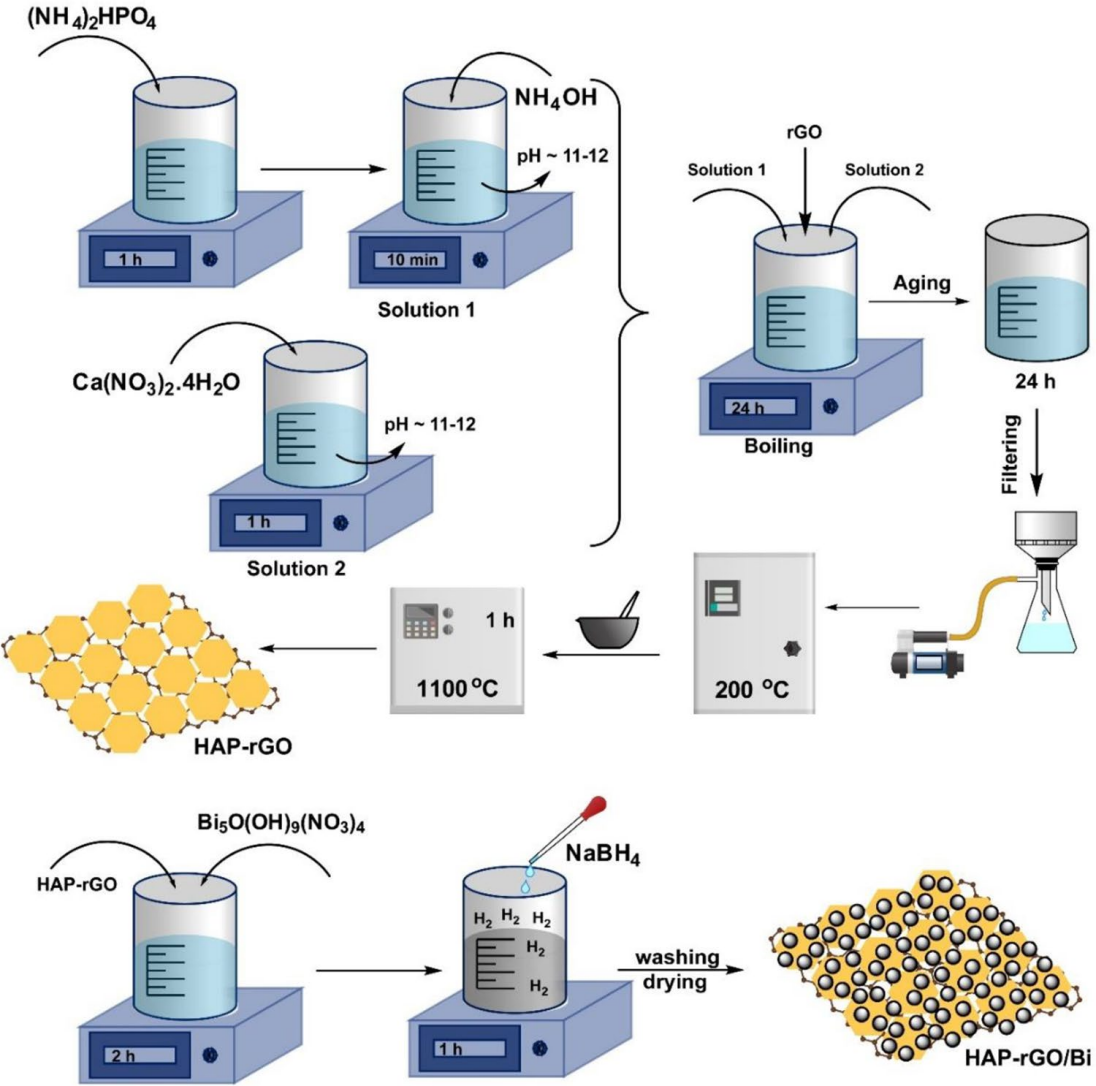
Schematic representation of Bi/HAp-rGO synthesis

To obtain the bismuth(0)-doped catalyst, the NaBH_4_ reduction method was used. Typically, a certain amount of HAp-rGO and bismuth(III) subnitrate (Bi_5_O(OH)_9_(NO_3_)_4_) was dispersed in 20 mL of ultra-pure water. The solution was mixed for 2 h. Then, NaBH_4_ was added dropwise to the above mixture, followed by stirring for another 1 h. After reaction completion, the obtained material was filtered and washed with ultra-pure water several times. Finally, it was dried in an oven at 70 °C overnight. The synthesis procedure is given schematically in Fig. 1.

### RSM-CCD-based catalytic reduction of MO

The decolorization of MO in the presence of NaBH_4_ was selected to investigate the catalytic activity of Bi/HAp-rGO. All the catalytic tests were achieved in a flask in which the volume of the solution was 5 mL and achieved under different conditions including time (min), MO concentration (Co, mg/L), NaBH_4_ amount (mM), and catalyst dosage (mg/mL). The detailed experimental conditions for the decolorization of MO are given in Table 1. The MO concentration after and before decolorization was measured using UV–Vis spectroscopy at λmax = 464 nm. The decolorization efficiency (%) was calculated by Eq. 1.

**Table 1:** CCD design matrix and results

Parameters	Units		Range and level		
			− 1	0	1
Time	min		0.5	2.75	5
C_o_	mg/L		5	27.5	50
NaBH_4_ amount	mM		1	15.5	30
Catalyst amount	mg/mL		0.5	2.75	5
Run	A	B	C	D	Decolorization efficiency, %
1	0.5	50	1	0.5	25.60
2	2.75	27.5	30	2.75	98.04
3	0.5	50	30	5	83.60
4	2.75	27.5	15.5	2.75	98.58
5	0.5	50	30	0.5	60.82
6	2.75	27.5	15.5	2.75	97.75
7	5	5	30	0.5	95.20
8	5	50	1	5	75.80
9	2.75	27.5	15.5	0.5	82.66
10	0.5	50	1	5	67.60
11	5	5	1	0.5	86.80
12	2.75	27.5	1	2.75	72.55
13	2.75	27.5	15.5	2.75	98.29
14	5	27.5	15.5	2.75	96.76
15	5	50	30	5	89.38
16	2.75	50	15.5	2.75	89.08
17	5	5	30	5	96.60
18	0.5	5	1	5	79.20
19	5	50	30	0.5	75.72
20	0.5	27.5	15.5	2.75	79.67
21	5	5	1	5	91.40
22	2.75	27.5	15.5	5	98.15
23	2.75	27.5	15.5	2.75	97.96
24	0.5	5	1	0.5	73.60
25	2.75	5	15.5	2.75	97.40
26	5	50	1	0.5	55.80
27	0.5	5	30	5	87.20
28	2.75	27.5	15.5	2.75	98.40
29	2.75	27.5	15.5	2.75	98.26
30	0.5	5	30	0.5	87.40

1 $$Decolorization \;efficiency \;\left(\%\right)=\frac{({C}_{o}-{C}_{e})}{{C}_{o}}\times \;100$$

The experimental design ensures simultaneous optimization of coefficients that affect the response, besides development performance features and minimizing errors with few tests. The RSM-CCD matrix was used to investigate and optimize the decolorization efficiency (%) of MO as a function of the parameters: time (min), MO concentration (Co, mg/L), NaBH_4_ amount (mM), and catalyst dosage (mg/mL). The total number of experiments was determined with the 2^ k^ + 2* k* + 6 equation (where *k* is the number of independent parameters). The six repetitions at the center point were performed to minimize experimental errors. A total of 30 experiments for the four parameters were performed to obtain decolorization efficiency (Table 1). The response for the optimization procedure can be acquired as quadratic or linear equations. The relation between the response and independent parameters can be given as the following equation.

2 $${y}_{p}={\beta }_{o}+\sum\limits_{i=1}^{k}{\beta }_{i}{x}_{i}+\sum\limits_{i=1}^{k}{\beta }_{ii}{x}_{i}^{2}+\sum\limits_{i=1}^{k}\sum\limits_{j=i+1}^{k}{\beta }_{ij}{x}_{i}{x}_{j}$$

where *y*_*p*_ is the response (decolorization efficiency); *x*_*i*_ and *x*_*j*_ are the independent variables; and *β0*, *βi*, *βii*, and *βij* are constant, linear, square, and interaction effect of the model, respectively. To confirm the suggested model’s statistical significance and practicality, analysis of variance (ANOVA) was employed. The significance of every parameter and the fit of the proposed model were explained by analyzing the correlation coefficient (*R*^2^), Fisher value (*F*-value), and probability value (*p*-value).

## Results and discussion

### Characterization

The surface morphology, elemental distribution on the surface, crystallographic properties, and oxidation state of Bi/HAp-rGO were investigated by SEM–EDX, elemental mapping, XRD, and XPS analysis.

**Fig. 2:**
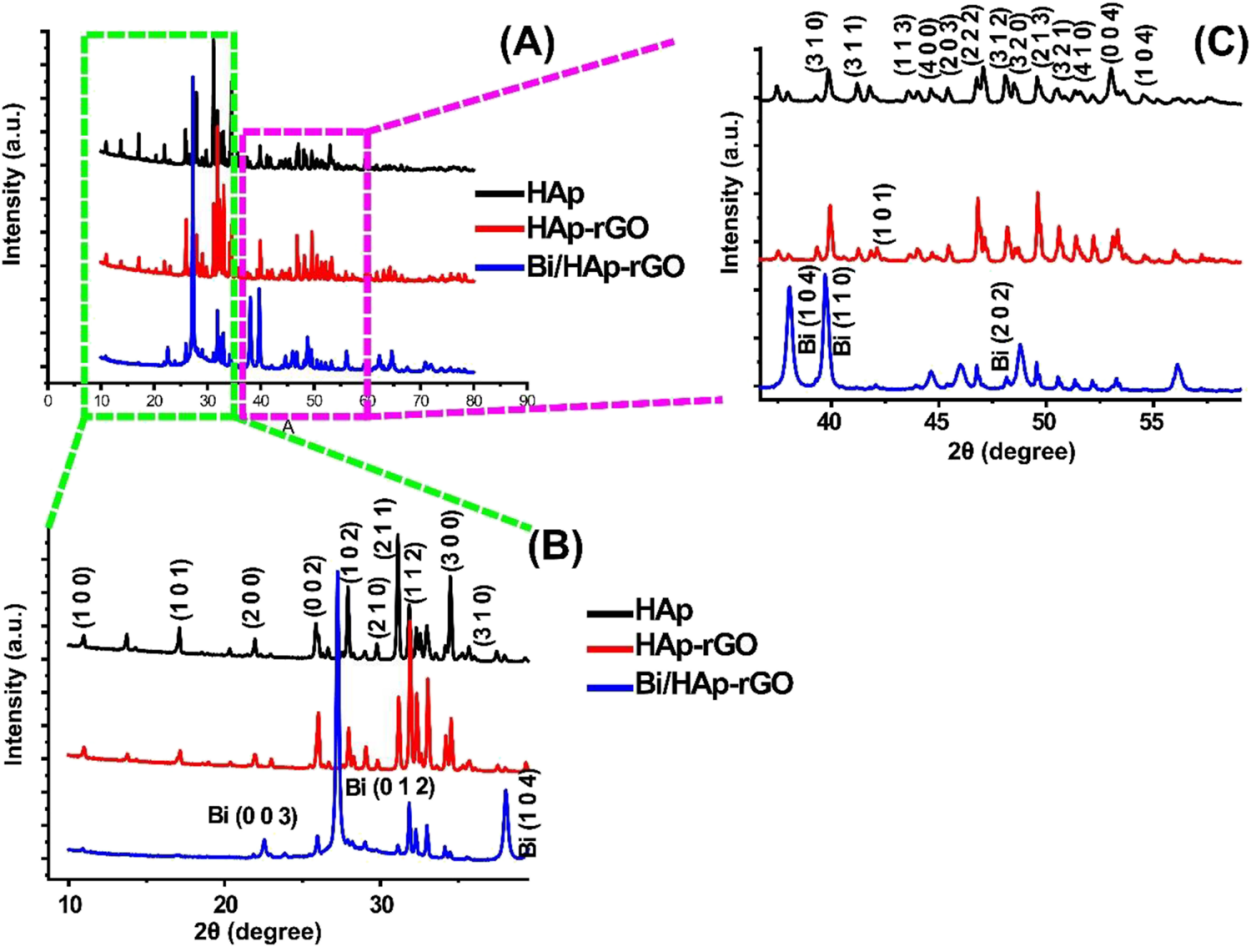
XRD pattern of Bi/HAp-rGO. **A** The entire spectrum. **B** 2*θ* range of 10–40°. **C** 2*θ* range of 40–60°

Figure 2 depicts the XRD pattern of Bi/HAp-rGO. The 10–40° and 40–60° 2*θ* ranges are magnified and shown in Fig. 2B, C. As can be clearly seen from Fig. 2B, C, almost all characteristic peaks of HAp are observed. The diffraction peaks observed at 25.9°, 31.1°, 31.9°, 32.9°, 34.4°, 39.8°, 46.7°, 49.5°, 50.5°, and 53.0° 2*θ* angles correspond to the reflection of the (002), (211), (112), (300), (202), (310), (222), (213), (321), and (004) planes of HAp, respectively (JCPDS card No: 09–432) (Rogina et al. 2013). Other characteristic peaks of HAp are labeled in Fig. 2B, C. Also, the peak at about 42.3° 2*θ* angle is attributed to the (101) facet of rGO (JCPDS card No: 41–1487) (Topçu and Dağcı Kıranşan 2019). The diffraction peaks detected at 22.6°, 27.3°, 38.1°, 39.7°, and 48.2° 2*θ* angles were attributed to the reflections of hexagonal Bi (003), Bi (012), Bi (104), Bi (110), and Bi (202) planes, respectively (JCPDS 86–1330) (Wang et al. 2007). As a result, almost all characteristic peaks of HAp, rGO, and Bi were observed, and it was concluded that Bi/HAp-rGO was successfully synthesized.

**Fig. 3:**
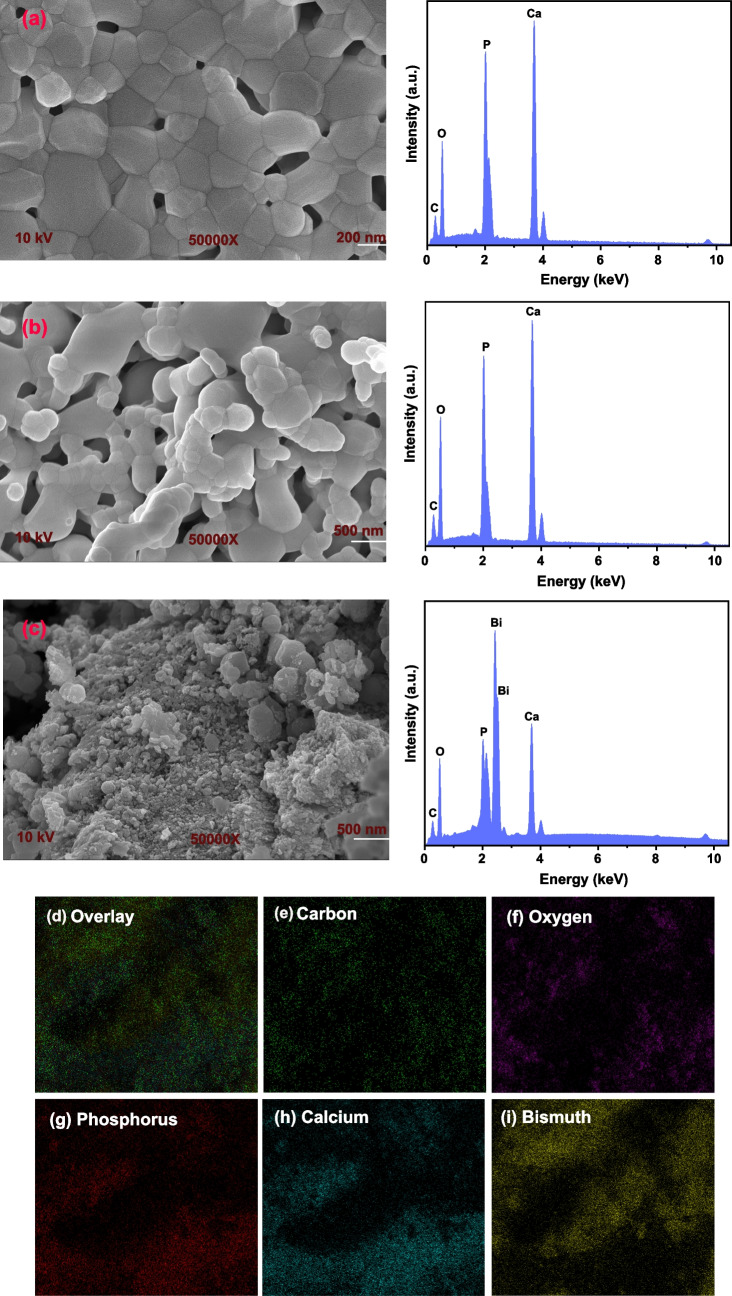
SEM images and corresponding EDX spectrum of **a** HAp, **b** HAp-rGO, and **c** Bi/HAp-rGO. Elemental mapping analysis of Bi/HAp-rGO for **d** overlay, **e** carbon, **f** oxygen, **g** phosphorus, **h** calcium, and **i** bismuth

SEM images and corresponding EDX spectra of HAp, HAp-rGO, and Bi/HAp-rGO are shown in Fig. 3. Figure 3a shows a conventional image of HAp that can be verified from the literature. The presence of P and Ca in the HAp structure was detected from the EDX spectrum. Although no significant changes are observed in the SEM images of HAp-rGO, the increase in the intensity of the oxygen peak in the EDX spectrum indicates that rGO has entered the structure (Fig. 3b). Bi nanoparticles reduced on HAp-rGO can be followed in Fig. 3c. Additionally, Bi peaks were detected in the EDX spectrum of Bi/HAp-rGO. Carbon and oxygen elemental mapping images of Bi/HAp-rGO indicate that rGO has a relatively more homogeneous distribution. Phosphorus and calcium elemental mapping images show that P and Ca in HAp are clustered in places. It was determined that the Bi distribution on HAp-rGO did not show severe agglomeration.

**Fig. 4:**
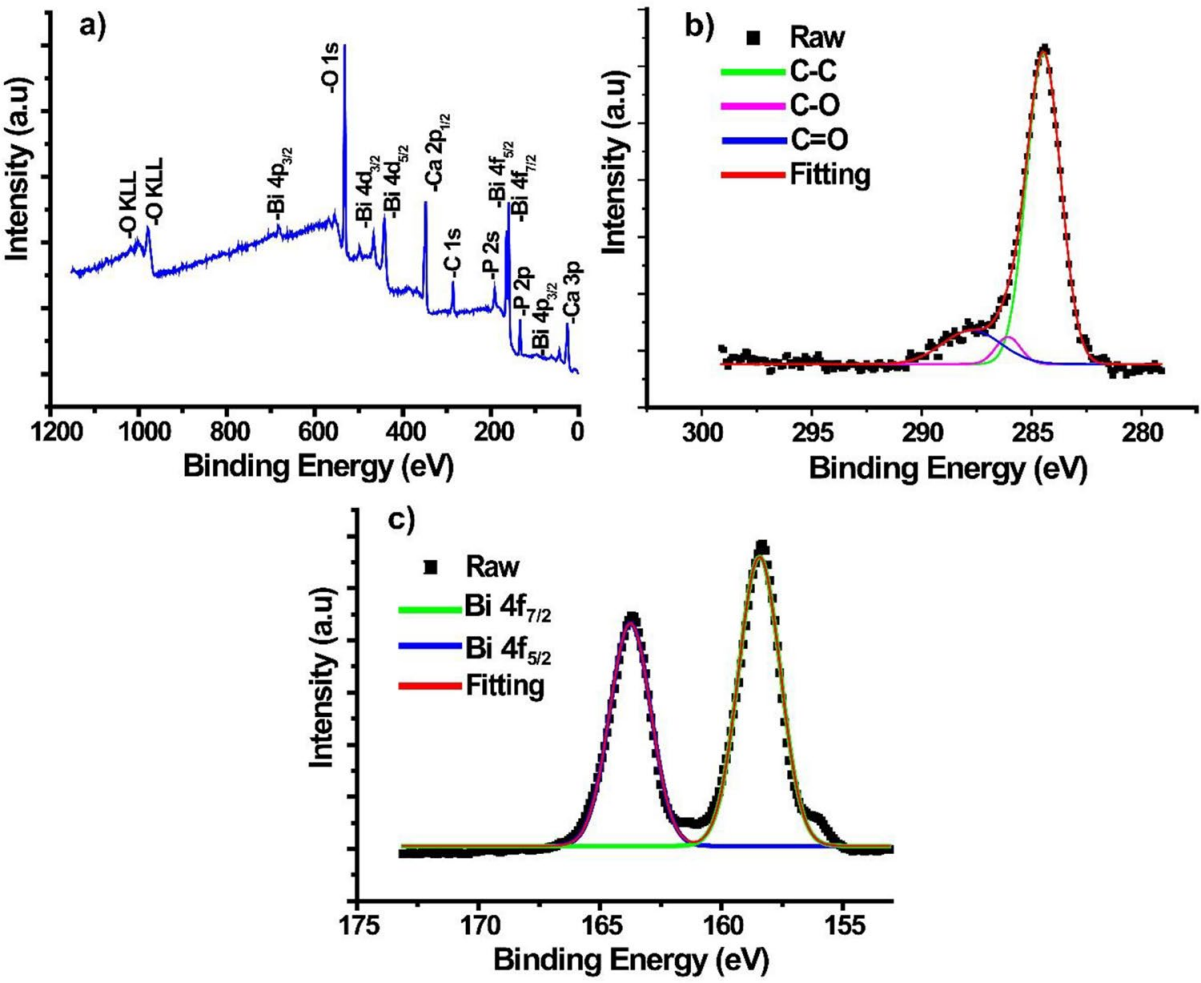
**a** General survey, **b** C 1 s, and **c** Bi 4f core level XPS spectra of Bi/HAp-rGO

XPS analysis was performed to determine the chemical state of the Bi/HAp-rGO catalyst. Figure 4 shows the general and partial XPS survey of Bi/HAp-rGO. In the general scanning spectrum of Bi/HAp-rGO, C 1 s and O 1 s arising from rGO and P 2 s, P 2p, Ca 2p, and Ca 3p peaks from HAp were observed (El-Aal et al. 2022; Zhang et al. 2015). In addition, Bi 4p, Bi 4d, Bi 5f, and Bi 4p peaks were detected indicating the presence of Bi in the catalyst system (Fig. 4a) (Rauf et al. 2015). The peaks at 284.5 eV, 286.1 eV, and 287.7 eV BE obtained as a result of the deconvolution of the C 1 s high-resolution spectra of Bi/HAp-rGO were attributed to sp^2^ carbon (C = C), C-O, and C = O, respectively (Qin et al. 2017). The relative C = C, C-O, and C = O ratios in the Bi/HAp-rGO structure were determined as 80.0%, 5.08%, and 14.9%, respectively. The convoluted C 1 s spectrum in Fig. 4b showed high compatibility with the spectra of rGOs reported in the literature (Johra and Jung 2015; Sharma et al. 2019). Bi 4f high-resolution spectra of Bi/HAp-rGO are given in Fig. 4c. The doublet detected at 163.7 eV and 158.5 eV BE was attributed to the presence of Bi 4f_5/2_ and Bi 4f_7/2_, respectively (Lu et al. 2019). From the Bi 4f core level spectra, it was seen that Bi in the Bi/HAp-rGO structure was in its elemental state. The absence of any oxide of Bi in the structure indicates that the Bi salt was successfully reduced in its elemental state onto HAp-rGO, and the XPS results support the XRD results in this respect.

### Statistical analysis based on RSM for MO decolorization

CCD in RSM was applied to evaluate the effects of the process parameters on the decolorization of MO via Bi/HAp-rGO in the existence of NaBH_4_. Four different parameters in three levels (*− 1*, *0*, + *1*)—time (A, min), MO concentration (C_o_) (B, mg/L), NaBH_4_ amount (C, mM), and catalyst dosage (D, mg/mL)—were tested for the selected response (the decolorization of MO, %). The obtained results are tabulated in Table 1.

A quadratic polynomial model expressing the relationship between the selected independent parameters and the response was presented in Eq. 2. ANOVA results for MO decolorization are represented in Table 2. It was reported that very high *F*-value and very low *p*-value (*p* < 0.05) were statistically significant for each parameter (Sharma et al. 2023; Zolfaghari et al. 2023). The low *p*-value of 0.0001 for the obtained model indicates that it is statistically significant. The significant model terms for MO decolorization were A, B, C, D, BC, BD, A^2^, and C^2^. Moreover, *R*^*2*^ value (*R*^2^ = 0.96) for MO decolorization is extremely high, indicating the applicability and adequacy of the created model.

**Table 2:** ANOVA results

Source	Sum of squares	df	Mean square	*F*-value	*p*-value	
Model	7507.75	14	536.27	27.76	< 0.0001	Significant
A-Time	783.70	1	783.70	40.57	< 0.0001	
B-C_o_	1632.11	1	1632.11	84.50	< 0.0001	
C-NaBH_4_ amount	1177.92	1	1177.92	60.98	< 0.0001	
D-Catalyst dosage	872.66	1	872.66	45.18	< 0.0001	
AB	16.97	1	16.97	0.88	0.3634	
AC	41.99	1	41.99	2.17	0.1610	
AD	58.22	1	58.22	3.01	0.1030	
BC	152.03	1	152.03	7.87	0.0133	
BD	473.50	1	473.50	24.51	0.0002	
CD	74.65	1	74.65	3.86	0.0681	
A^2^	91.15	1	91.15	4.72	0.0463	
B^2^	2.14	1	2.14	0.11	0.7437	
C^2^	203.32	1	203.32	10.53	0.0054	
D^2^	36.42	1	36.42	1.89	0.1899	
*R*^2^ = 0.9628						

3 $$\begin{array}{l}The \;decolorization \;of \;MO\left(\mathrm{\%}\right)=+62.55557+10.62281\left[Time\right]-0.82231[{C}_{o}]\\ +1.92283\left[{NaBH}_{4} \;amount\right]+6.27541\left[Catalyst \;dosage\right]+0.020346\left[Time\right][{C}_{o}]\\ \begin{array}{l}-0.049655\left[Time\right]\left[{NaBH}_{4} \;amount\right]-0.37679[Time][Catalyst \;dosage]\\ +9.44828E-003\left[{C}_{o}\right]\left[{NaBH}_{4} \;amount\right]+0.10746[{C}_{o}][Catalyst \;dosage]\\ \begin{array}{l}-0.066207\left[{NaBH}_{4} \;amount\right]\left[Catalyst \;dosage\right]-1.17161{\left[Time\right]}^{2}-1.79649E-003{[{C}_{o}]}^{2}\\ -0.042133{\left[{NaBH}_{4} \;amount\right]}^{2}-0.74064{[Catalyst \;dosage]}^{2}\end{array}\end{array}\end{array}$$

**Fig. 5:**
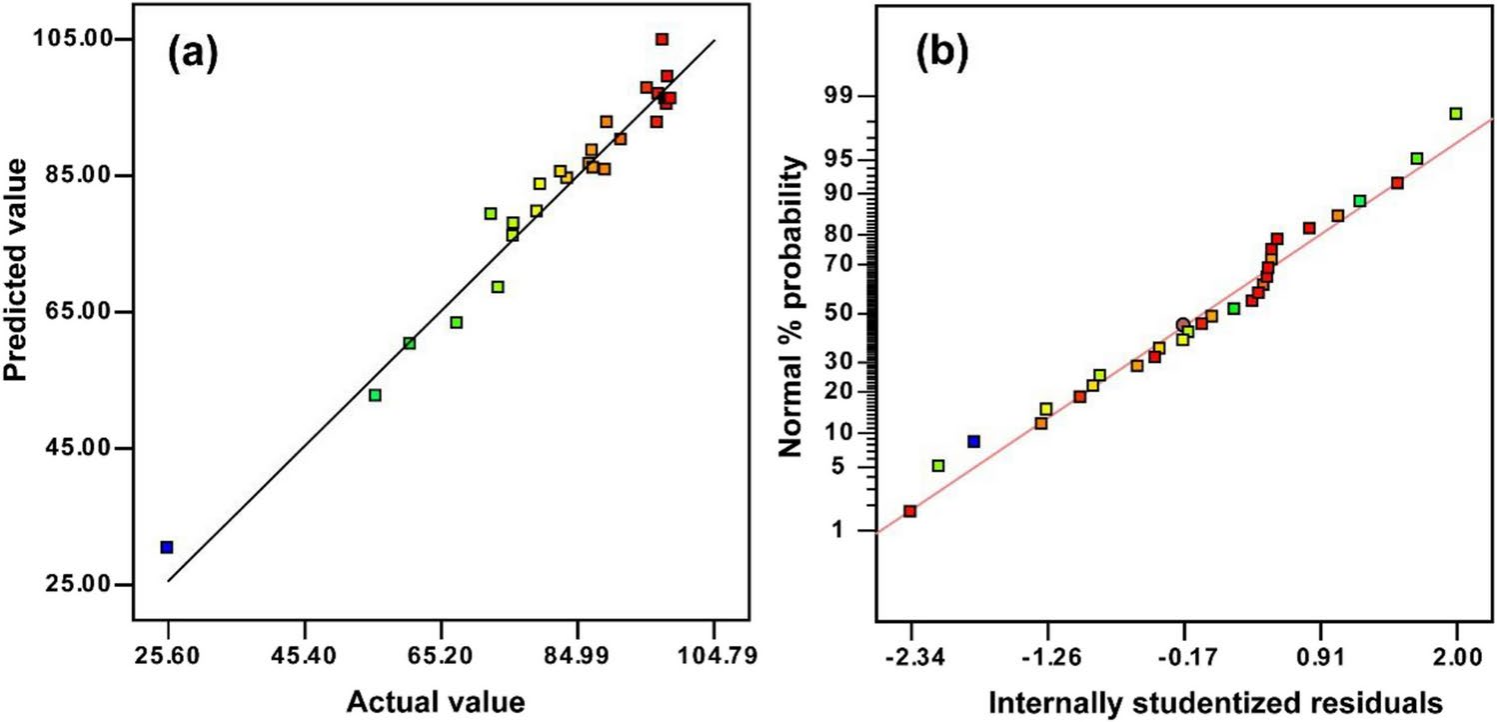
**a** Predicted versus actual plot and **b** normal probability plot obtained for MO decolorization

The plot of the estimated responses by the model versus actual responses is demonstrated in Fig. 5a. As seen in Fig. 5a, the data is normally distributed on a straight line, confirming the model’s unique ability to predict experimental data. On the other hand, a normal probability plot of the residuals is given in Fig. 5b. From Fig. 5b, the residuals are dispersed around a straight line. This statistical analysis shows that the model is applicable. According to the ANOVA results and Fig. 5, it can be said that the suggested model is valid for the system.

**Fig. 6:**
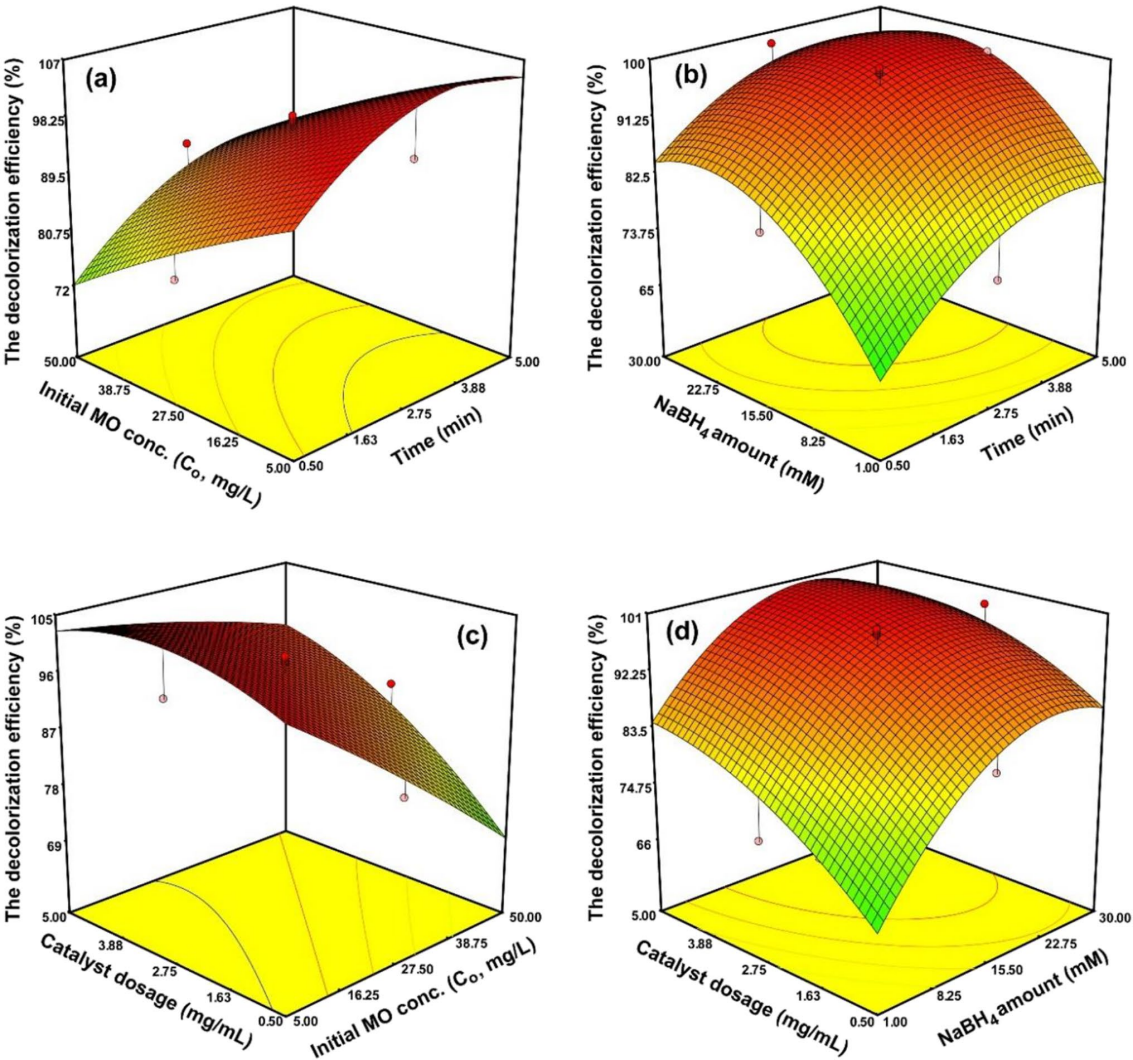
3D response plots of **a** time, C_o_; **b** time, NaBH_4_ amount; **c** C_o_, catalyst dosage; and **d** NaBH_4_ amount, catalyst dosage

3D RSM plots to investigate the effects of each independent parameter and determine the optimal conditions for maximum response were generated in Fig. 6. The reaction time shows a noticeable effect in the decolorization efficiency of MO dye. With an increase in reaction time, more decolorization was obtained. The decolorization efficiency of MO due to the increase in reaction time is seen in Fig. 6a, b. This is due to the reaction time between the synthesized catalyst and MO dye affecting the saturation state of the Bi/HAp-rGO surface. With the increase of reaction time, MO molecules will interact more with the reaction sites on the catalyst surface, resulting in increased decolorization efficiency of MO (Al-Ansari et al. 2024; Zhou et al. 2024). It was observed that the maximum decolorization efficiency of MO reached its maximum in about 3 min and did not show a significant increase at later levels. Figure 6a, c shows the effect of C_o_ on the MO decolorization efficiency. The results revealed that increasing the concentration of MO dye from 5 to about 19 mg/L partially increased the MO decolorization efficiency. It was observed that the decolorization efficiency gradually decreased at higher concentrations. The positive effect on decolorization efficiency observed at low C_o_ values is initially attributed to the fact that fewer MO molecules move freely to more active sites on Bi/HAp-rGO (Shrivastava et al. 2024). In addition, the decrease in decolorization efficiency at high levels indicates that more MO molecules block the active sites of the catalyst, and therefore, the efficiency decreases due to the competition of more MO dyes on the catalyst surface (Roy et al. 2023; Shrivastava et al. 2024). Moreover, the MO and BH_4_^−^ ions on the surface of Bi/HAp-rGO were adsorbed, based on the Langmuir–Hinshelwood process. This is a reversible process that creates competition between both MO and BH_4_^−^ for the active sites of Bi/HAp-rGO (Anwar et al. 2021). For this reason, it can be stated that a high concentration of MO may reduce the yield by slowing down the reaction rate.

The amount of NaBH_4_ was varied within the range of 1 to 30 mM to explore the impact on the decolorization activity. Figure 6b, d shows that the effect of the amount of NaBH_4_ on the decolorization efficiency increased up to about 20 mM beyond which a gradual decrease was noticed due to the increase in NaBH_4_ amount. As the amount of NaBH_4_, a strong reducing agent, increased, more hydrogen was produced and bound to the catalyst and the decolorization efficiency of the dye increased. This can be attributed to BH_4_^−^ ions as a result of the ionization of NaBH_4_. Through the catalyst, which functions as an electron carrier, hydrogen interacts with the MO molecules, reducing them (Acar et al. 2023). Also, this suggests that electron donor BH_4_^−^ is delivered to the catalyst and electrons are transported to acceptor MO molecules, resulting in the decolorization of the dye molecules (Alshaikhi et al. 2022). On the other hand, the use of excessive amounts of NaBH_4_ prevented dye decolorization because when the catalyst amount was constant, the produced hydrogen adhered to the catalyst surface and the active sites required on the catalyst surface for the adsorption of MO molecules decreased and as a result, the efficiency was likely to decrease (Naseem et al. 2019). As displayed in Fig. 6c, d, there was a positive impact of Bi/HAp-rGO on the decolorization of MO when the catalyst dosage increased from 0.5 to 5 mg/mL, and it reached a maximum of about 3 mg/mL. However, the catalyst dosage had no significant change on the decolorization efficiency when the catalyst dosage was high enough. The increase of MO decolorization efficiency with increasing Bi/HAp-rGO dosage could be due to more available active sites, resulting in more dye to be decolored (Saikia et al. 2017; Wang et al. 2020).

### Optimization and validation stage

The decolorization of MO dye using Bi/HAp-rGO in the presence of NaBH_4_ was optimized using RSM modeling via the Design-Expert program (trial version). Four major parameters (time, C_o_, NaBH_4_ amount, and Bi/HAp-rGO dosage) were selected for the optimization of MO decolorization, while the decolorization efficiency of MO as response. The optimum conditions of the independent parameters and response were obtained by setting the parameters to “in range” and the responses to “maximum.” In the optimization stage, the desirability function, which ranges from 0 (unfavorable response) to 1 (favorable response), is a numerical tool used to determine the desired target for both the independent parameters and the response (Zolfaghari et al. 2023). The optimal conditions for the decolorization of MO using Bi/HAp-rGO in the existence of NaBH_4_ were determined by selecting the best desirability function by the software. The optimum points were found as time, 2.91 min; C_o_, 18.85 mg/L; NaBH_4_ amount, 18.35 mM; and Bi/HAp-rGO dosage, 2.12 mg/mL. At the determined process conditions, the maximum decolorization efficiency of MO was found to be 99.60% with an overall desirability of 1. The validation of the suggested model was carried out by keeping the parameters at the obtained optimal points, and the decolorization efficiency of MO was experimentally 99.49%. The negligible difference between the experimental and predicted results indicated that the suggested model can be acceptable.

At the achieved optimal conditions, further experiments were conducted to evaluate the significance of Bi/HAp-rGO. For this purpose, the effects of HAp, HAp-rGO, only NaBH_4_, and Bi/HAp-rGO without NaBH_4_ were investigated on the decolorization efficiency of MO (Fig. S1). The decolorization efficiencies of MO for HAp, HAp-rGO, only NaBH_4_, and Bi/HAp-rGO without NaBH_4_ were found as 33.1%, 41.91%, 16.45%, and 18.24%, respectively, at the optimal process conditions. A slight change in the decolorization percentage of MO was observed in the absence of either NaBH_4_ or catalyst. However, it was observed that the addition of both catalyst and NaBH_4_ increased dye decolorization, indicating that the catalyst in the existence of NaBH_4_ has a synergistic effect on the decolorization of MO (Ravikumar et al. 2016). Therefore, it clearly demonstrated that neither the catalyst nor NaBH_4_ alone was suitable to finish the rapid decolorization of MO. On the other hand, the decolorization efficiency of Bi/HAp-rGO is quite high compared to HAp and HAp-rGO materials. So, it is possible to conclude that the catalyst effectively increased the decolorization of MO by transmitting electrons from BH_4_^−^ species to MO through the Bi NPs (Saikia et al. 2017). These results suggested that the Bi/HAp-rGO is effective as a catalyst in the presence of NaBH_4_ for the decolorization of MO.

The catalytic activity of the Bi/HAp-rGO for the decolorization of MO was compared with various catalysts from the literature given in Table 3. By comparison, Bi/HAp-rGO demonstrated higher or similar catalytic activities compared to the various catalyst systems reported in the literature for MO decolorization. Furthermore, the present work provides a new perspective on the application of the optimization process for both the effective usage of catalysts and the catalytic reduction of dyes. This shows that the study will significantly add to the literature and can be a reference for future studies.

**Table 3:** Comparison of various catalysts for the decolorization of MO

Catalyst	Time	C_o_	NaBH_4_ amount	Catalyst amount	Decolorization efficiency (%)	Ref
Pd NPs/RGO-*A. abrotanum*	140 s	10 mg/L	0.1 M	0.33 mg/mL	99	(Hashemi Salehi et al. 2019)
CoFe_2_O_4_/γ-Fe_2_O_3_	36 min	50 mg/L	0.3 M	2 mg/mL	≥ 99.9%	(El-Subruiti et al. 2019)
Fe_3_O_4_@Nico-Ag	12 min	10 mM	100 mM	0.33 mg/mL	~ 100	(Kurtan et al. 2016)
Pd/CNFs	240 min	10 mg/L	0.026 mM	2 mg/mL	98.9	(Najem et al. 2020)
Au/Fe_3_O_4_-chitosan	18 min	0.1 mM	0.04 M	2 mg/mL	> 90	(Liu et al. 2016)
SiO_2_-Ag CS Nps	5 min	0.0524 mM	0.001 M	0.5 mL	91.7	(Khalik et al. 2020)
Bi/HAp-rGO	2.91 min	18.85 mg/L	18.35 mM	2.12 mg/mL	99.6	This study

### Kinetic and thermodynamic studies

The pseudo-first-order kinetic model could be evaluated for the decolorization of MO by Bi/HAp-rGO in the existence of the high concentration of the reducing agent (Sarkar et al. 2021). The pseudo-first rate constant (*k*_app_) was calculated by using the pseudo-first-order kinetic model equation (Eq. S1). The kinetic plot of ln(C_o_/C_t_) vs. reaction time at different temperatures is shown in Fig. S2a. The results obtained from the kinetic model are presented in Table S1. The results showed that the *k*_app_ was found to increase with temperature. The Arrhenius equation (Eq. S2) can be used to compute the reaction activation energy (*E*_a_). The value of *E*_a_ was calculated from the linear correlation slope between lnk versus 1/T (Fig. S2b). This value was calculated to be 6.75 kJ/mol for the decolorization of MO by Bi/HAp-rGO in the existence of NaBH_4_. The activation enthalpy (Δ*H*^#^) and activation entropy (Δ*S*^#^) as thermodynamic parameters for the decolorization of MO were calculated from the linear plot of lnk/T vs. 1/T (Fig. S2c), and the values are presented in Table S1. The Δ*H*^#^ value obtained in the case of the decolorization of MO was 4.19 kJ/mol, indicating that there is an endothermic nature for the decolorization process due to the positive value of its. Moreover, the value of Δ*S*^#^ was found to be − 223.36 J/mol K. This negative value indicated the decrease in the randomness at reaction solution interfaces via decolorization of MO, namely, lower randomness (Akl et al. 2023; Srivastava et al. 2022).

### MO decolorization mechanism of Bi/HAp-rGO

**Fig. 7:**
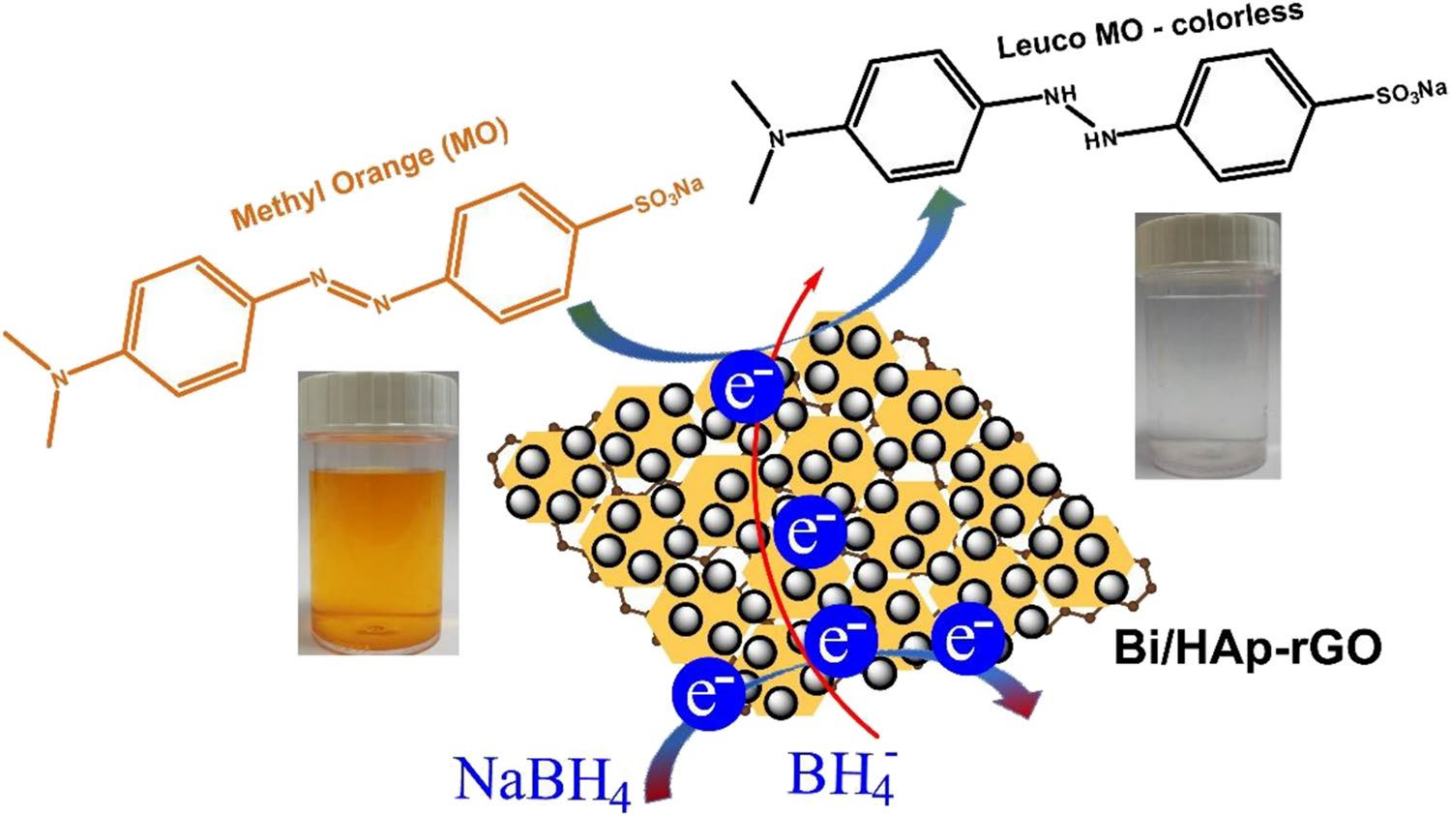
The possible decolorization mechanism of MO on Bi/HAp-rGO

The possible mechanism for the decolorization of MO under the optimal conditions using Bi/HAp-rGO catalyst is shown in Fig. 7. NaBH_4_ as a reducing agent was used to the decolorization of MO molecules in aqueous settings. In general, MO aqueous solution is orange. However, in the existence of a reducing agent (NaBH_4_), the MO dye molecules were turned into a colorless form (Leuco-MO). Bi NPs serve as an electron transfer mechanism for MO dye, transferring electrons from an electron donor to an electron acceptor. BH_4_^−^ ions generated from NaBH_4_ act as electron donors (nucleophilic), while MO molecules as electrophile (electron acceptor) (Mascarenhas & Varanda 2021). In the first stage, simultaneous adsorption of BH_4_^−^ ions and MO dye onto the catalyst surface occurs. Later, electron transfer occurs between nucleophilic BH_4_^−^ ions and the electrophilic MO molecules through Bi/HAp-rGO. Finally, the orange color MO becomes colorless (Keypour et al. 2023; Truong et al. 2024). In general, NaBH_4_, a reducing agent, is decomposed as H_2_, diborane, and electrons. H_2_ and electrons are captured by the catalyst (Mascarenhas and Varanda 2021). Then, adsorption of the H_2_ molecule and subsequent dissociation onto the Bi NPs surface occurs. Bi NPs produce partly charged H_2_ species on their surface by functioning as an electron relay. MO molecules are adsorbed to the surface of the catalyst. The adsorbed MO molecules are reduced via electron capture of the active sites of the catalyst along with H_2_ species (Hata et al. 2022, Mascarenhas and Varanda 2021).

## Conclusion

The optimization and modeling of MO decolorization in the presence of NaBH_4_ were applied experimentally via RSM-CCD. An easy approach was developed to produce a Bi/HAp-rGO as a catalyst for the decolorization of MO. The functional relation among the decolorization efficiency and four parameters (time, MO conc., NaBH_4_ amount, and catalyst amount) was defined using the obtained quadratic model. The optimum conditions for MO decolorization were time 2.91 min, MO conc. 18.85 mg/L, NaBH_4_ amount 18.35 mM, and Bi/HAp-rGO dosage 2.12 mg/mL. Almost all of the MO molecules were observed to be decolorized under the conditions obtained (99.6%). Lastly, a possible mechanism for decolorizing MO was put forth using Bi/HAp-rGO in the presence of NaBH_4_. To summarize, it can be said that Bi/HAp-rGO shows great promise as a material for the highly effective removal of contaminants in water treatment.

### Supplementary Information

Below is the link to the electronic supplementary material.

Supplementary file1 (DOCX 513 KB)

## Data Availability

The authors confirm that the data supporting the findings of this study are available within the article.
