# Supplementary material for: Optimization of methyl orange decolorization by bismuth(0)-doped hydroxyapatite/reduced graphene oxide composite using RSM-CCD
Source: Environ Sci Pollut Res Int. 2024 Apr 27;31(23):33371–84. doi: 10.1007/s11356-024-33425-4 (PMC11136733; doi:10.1007/s11356-024-33425-4)
Supplement: Supplementary file 1 — Supplementary file1 (DOCX 513 KB) [file 11356_2024_33425_MOESM1_ESM.docx]

**Supplementary Material**

**Optimization of Methyl Orange Decolorization by Bismuth (0)-Doped Hydroxyapatite/Reduced Graphene Oxide Composite Using RSM-CCD**

Umit Ecer^1*^, Sakir Yilmaz,^1,2^, Berdan Ulasa, ^1,2^, Serap Koc^3^

^1^ Department of Chemical Engineering, Institute of Natural and Applied Sciences, Van Yuzuncu Yil University, 65080, Van, Turkey

^2^ Department of Mining Engineering, Faculty of Engineering, Van Yuzuncu Yil University, Van, 65000, Turkey

^3^ Department of Mechanical Engineering, Faculty of Engineering, Van Yuzuncu Yil University, Van, 65000, Turkey

* Corresponding Author: umitecer@gmail.com


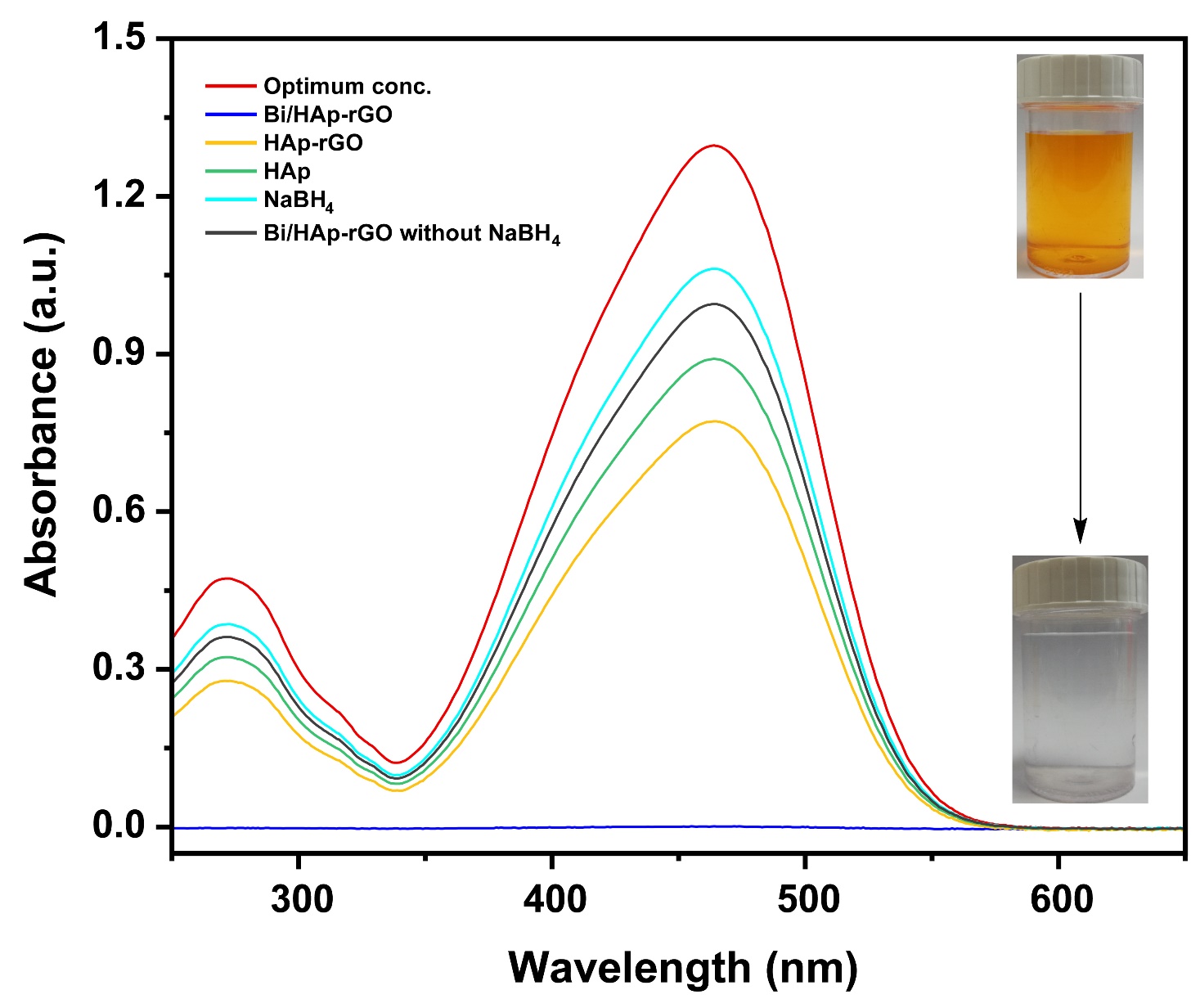


**Fig. S1.** UV-vis absorption spectra for the decolorization efficiency of MO at the optimal conditions.


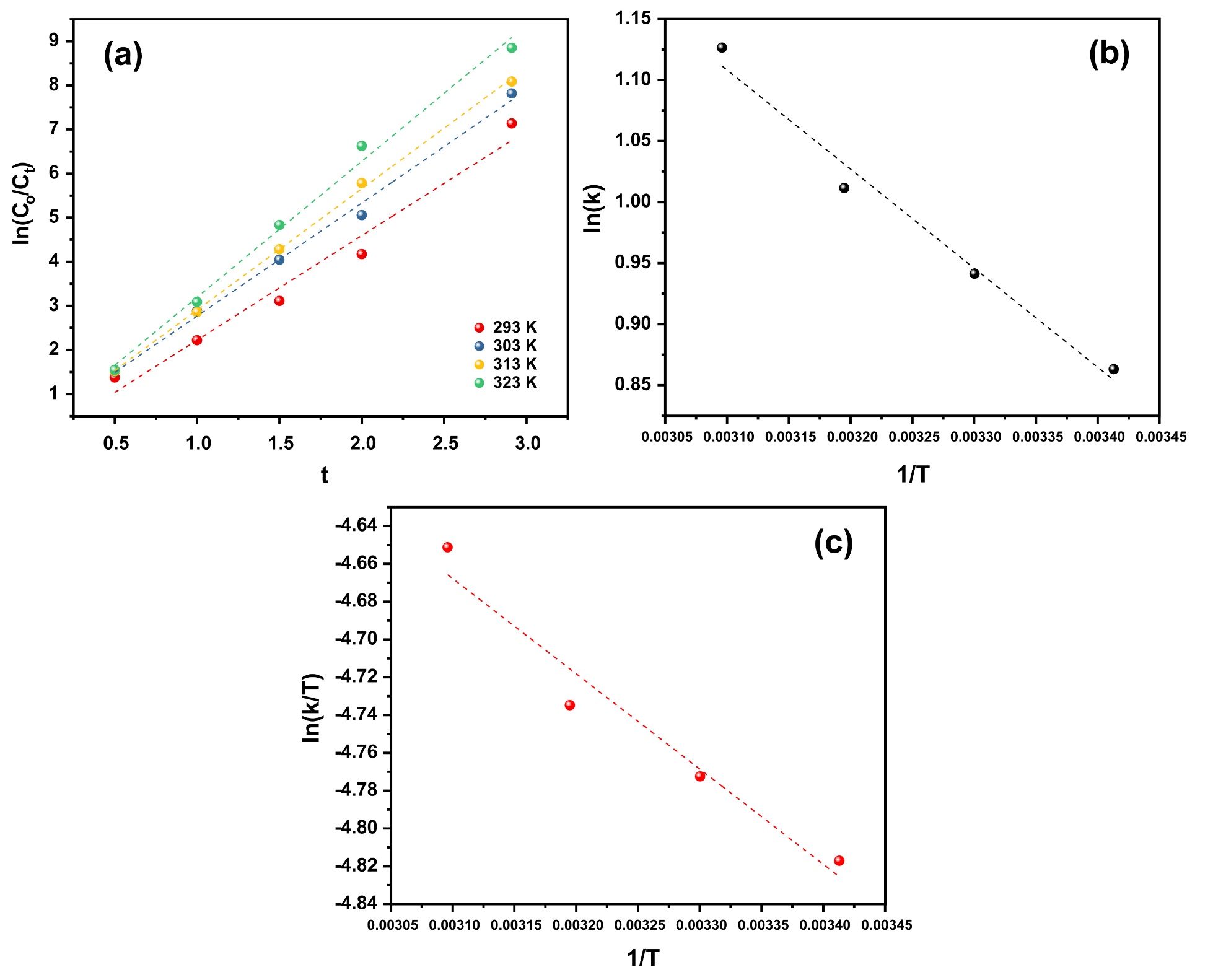


**Fig. S2.** (a) ln(C_o_/C_t_) versus t plot, (b) lnk versus 1/T for the decolorization of MO, and (c) lnk/T versus 1/T for the decolorization of MO.

| $\ln\frac{C_{o}}{C_{t}}=k_{app}t$ | (S1) |
| --- | --- |
|  | (S2) |
| $\ln\frac{k}{T}=ln\frac{k_{B}}{h}+\frac{\Delta S^{\#}}{R}-\frac{\Delta H^{\#}}{RT}$ | (S3) |

where *C_o_* and *C_t_* are the concentration of MO at initial time and time *t*, respectively (mg/L). *k_app_* is the pseudo-first rate constant of MO decolorization (min^-1^). *E_a_* is the activation energy for the decolorization of MO (J/mol). *T* is the absolute temperature (K). *R* is the universal gas constant (8.314 J/mol K). *A* is Arrhenius factor, *k_B_* is the Boltzmann constant (1.381×10^-23^ J/K), *h* is the Planck constant (6.626×10^-34^ J/mol K), *ΔS^#^* is the activation entropy (J/mol K), and *ΔH^#^* is the activation enthalpy (J/mol).

**Table S1** Kinetic and thermodynamic parameters of MO decolorization.

| *T* (K) | *k* (min^-1^) | *E_a_* (kJ/mol) | *ΔS^#^* (J/mol K) | *ΔH^#^* (kJ/mol) |
| --- | --- | --- | --- | --- |
| 293 | 2.37 | 6.75 | -223.36 | 4.19 |
| 303 | 2.56 |  |  |  |
| 313 | 2.75 |  |  |  |
| 323 | 3.08 |  |  |  |
